# Supplementary material for: Compulsive and compensative buying among online shoppers: An empirical study
Source: PLoS One. 2021 Jun 3;16(6):e0252563. doi: 10.1371/journal.pone.0252563 (PMC8174690; doi:10.1371/journal.pone.0252563)
Supplement: S1 File — (PDF) [file pone.0252563.s001.pdf]

800
2019-03-27

Base: all respondents

S02 [S] **Czy w ciągu ostatniego roku kupił(a) Pan(i) jakikolwiek produkt/ usługę przez internet?**
1. TAK
2. NIE

Base: respondents who bought something online – code 1 in Q02

S03 [S] **Które z poniższych rodzajów produktów/ usług kupił(a) Pan(i) przez internet w ciągu ostatniego roku przynajmniej 1 raz?**
ANK.: POKAŻ EKRAN
1. Artykuły spożywcze
2. Artykuły kosmetyczne/ perfumeryjne
3. Odzież
4. Obuwie
5. Akcesoria (paski, portfele)
6. Mały sprzęt gospodarstwa domowego (odkurzacze, suszarki, żelazka, roboty kuchenne, itp.)
7. Duży sprzęt gospodarstwa domowego (pralki, zmywarki, piekarniki, płyty kuchenne, itp.)
8. Telewizory
9. Komputery
10. Meble
11 Artykuły do urządzania mieszkania
12. Artykuły do ogrodu
13. Artykuły sportowe
14. Książki,
15. Muzyka (np. Spotify, Tidal)
16. Telewizja na żadanie (Netflix, VOD, Showmax, filmy, itp.)
17. Gry (komputerowe i na konsole)
18. Artykuły dla dzieci
19. Zabawki
20. Jedzenie na wynos
21. Bilety (podróże)
22. Bilety rozrywka (koncerty, kino)
23. Noclegi
24. Usługi, np. przedpłacone karty, usługi telekomunikacyjne

Base: respondents who bought something online – code 1 in Q02

P1 [S in each row] **Jak często kupuje Pan(i) produkty/ usługi z różnych kategorii w następujących lokalizacjach?**
ANK.: POKAŻ EKRAN
SCRIPTER: SHOW ONLY PRODUCTS/ SERVICES MENTIONED IN Q03
In row:
1. Artykuły spożywcze
2. Artykuły kosmetyczne/ perfumeryjne
3. Odzież
4. Obuwie
5. Akcesoria (paski, portfele)
6. Mały sprzęt gospodarstwa domowego (odkurzacze, suszarki, żelazka, roboty kuchenne, itp.)
7. Duży sprzęt gospodarstwa domowego (pralki, zmywarki, piekarniki, płyty kuchenne, itp.)
8. Telewizory
9. Komputery
10. Meble
11. Artykuły do urządzania mieszkania
12. Artykuły do ogrodu
13. Artykuły sportowe
14. Książki,
15. Muzyka (np. Spotify, Tidal)
16. Telewizja na żadanie (Netflix, VOD, Showmax, filmy, itp.)

17. Gry (komputerowe i na konsole)
18. Artykuły dla dzieci
19. Zabawki
20. Jedzenie na wynos
21. Bilety (podróże)
22. Bilety rozrywka (koncerty, kino)
23. Noclegi
24. Usługi, np. przedpłacone karty, usługi telekomunikacyjne

In column:
1. Specjalistyczne sklepy internetowe oferujące produkty tylko jednej marki
2. Sklepy internetowe oferujące produkty wielu marek
3. Allegro
4. OLX
5. Ali Express
6. Sklepy w centrach handlowych
7. Sklepy wolnostojące poza centrami handlowymi
8. Targ, bazar, handel obwoźny

1. Raz w tygodniu lub częściej
2. 2-3 razy w miesiącu
3. Raz w miesiącu
4. Kilka razy w roku
5. 1-2 razy w roku
6. Rzadziej
7. Nigdy

Base: respondents who bought something online – code 1 in Q02
P19 [S]
Proszę teraz porównać Pana(i) przeciętne wydatki na zakupy w internecie i w sklepach stacjonarnych. Które z następujących stwierdzeń najlepiej opisuje Pana(i) sytuację?
1. Zdecydowanie więcej wydaję na zakupy przez internet niż na zakupy w sklepach stacjonarnych
2. Trochę więcej wydaję na zakupy przez internet niż na zakupy w sklepach stacjonarnych
3. Mniej więcej wydaję tyle samo na zakupy przez internet co na zakupy w sklepach stacjonarnych
4. Trochę więcej wydaję na zakupy w sklepach stacjonarnych niż na zakupy przez internet
5. Zdecydowanie więcej wydaję na zakupy w sklepach stacjonarnych niż na zakupy przez internet

Base: all respondents
P20 [S in each row]
W jakim stopniu zgadza lub nie zgadza się Pan(i) z następującymi stwierdzeniami dotyczącymi zakupów?
ANK.: POKAŻ EKRAN
In row:
1. Kupuję w internecie, by zrobić na znajomych i przyjaciołach wrażenie jakimś fajnym zakupem
2. Kupowanie przez internet stało się teraz bardzo modne
3. Internet jest dla mnie najdogodniejszym sposobem kupowania różnych usług i produktów.
4. Internet służy mi głównie jako źródło informacji, a zakupów w większości przypadków dokonuję w sklepach tradycyjnych.
5. Bardzo często najpierw oglądam produkty w sklepie tradycyjnym, a potem kupuję je w Internecie.
6. Nie jestem entuzjastą zakupów w internecie, wolę zakupy tradycyjne
7. Nie lubię robić zakupów ani online ani w sposób tradycyjny
8. Wolę zakupy w tradycyjnych sklepach – przynajmniej można wyjść z domu i zażyć trochę ruchu

In column:
1. W ogóle się nie zgadzam
2. Raczej się nie zgadzam
3. Ani się zgadzam, ani nie zgadzam
4. Raczej się zgadzam
5. Zgadzam się całkowicie

Base: all respondents

P22 [S in each row]

Przeczytam teraz Panu(i) kilka stwierdzeń, opisujących zachowania zakupowe różnych ludzi. Po odczytaniu każdego stwierdzenia proszę powiedzieć, w jakim stopniu zgadza albo nie zgadza się Pan(i) z tym stwierdzeniem?

ANK: POKAŻ EKRAN I ODCZYTAJ

SCRIPTER ROTATION

In rows:

1. Czasami bez szczególnej przyczyny nagle czuję, że muszę wyjść z domu i pójść na zakupy
2. Czasami mam wyrzuty sumienia, gdy coś sobie kupię
3. Czasami widzę coś i czuję, że muszę to kupić
4. Czasami zauważam, że coś mnie popycha do tego, by iść na zakupy
5. Często kupuję coś tylko dlatego, że jest tanie
6. Często kupuję coś, gdyż po prostu mam ochotę na kupowanie
7. Często mam poczucie, że jakąś rzecz muszę bezwzględnie mieć
8. Często nie mam odwagi pokazać kupionych rzeczy innym osobom, gdyż mogliby mnie uznać za osobę nierozsądną
9. Często po zakupie jakiejś rzeczy pytam samego siebie, czy rzeczywiście ten zakup był taki ważny
10. Gdy idę przez miasto albo przez centrum handlowe/ sklep, czuję silną potrzebę, by coś sobie kupić
11. Gdy mam pieniądze, czuję, że muszę je wydać
12. Jestem raczej rozrzutny(a)
13. Katalogi sprzedaży wysyłkowej/ strony sklepów internetowych są dla mnie dużą pokusą, po ich obejrzeniu najczęściej wysyłam zamówienie
14. Robię zakupy, by uciec od niemiłej codzienności i się zrelaksować
15. Zdarzało mi się często kupić coś, czego potem w ogóle nie używałe(a)m
16. Zdarzało mi się często kupić coś, na co właściwie w ogóle nie mogłe(a)m sobie pozwolić

In columns:

1. W ogóle nie zgadzam się
2. Raczej nie zgadzam się
3. Raczej zgadzam się
4. Zgadzam się całkowicie
