# Supplementary material for: Compulsive and compensative buying among online shoppers: An empirical study
Source: PLoS One. 2021 Jun 3;16(6):e0252563. doi: 10.1371/journal.pone.0252563 (PMC8174690; doi:10.1371/journal.pone.0252563)
Supplement: S2 File — (PDF) [file pone.0252563.s002.pdf]

800
2019-03-27

Base: all respondents

S02 [S] **Did you buy a product or a service online in the last year?**
1. YES
2. NO

Base: respondents who bought something online – code 1 in S2

S03 [S] **Which of the following kinds of products/ services did you buy online in the last year at least once?**
INT.: SHOW THE SCREEN
1. Groceries
2. Cosmetics
3. Clothing
4. Footwear
5. Leather accessories
6. Small household appliances (vacuum cleaners, dryers, irons, food processors etc.)
7. Big household appliances (washing machines, dishwashers, ovens etc.)
8. TV sets
9. Computers
10. Furniture
11. Interior design articles
12. Garden articles
13. Sport articles
14. Books
15. Music (e.g. Spotify, Tidal)
16. Payable TV (Netflix, VOD, Showmax etc.)
17. Games (for PCs, consoles)
18. Articles for children
19. Toys
20. Dishes takeaway
21. Tickets (travels)
22. Entertainment tickets (concerts, cinema)
23. Accommodation
24. Services, e.g. pre-paid cards, telecommunication services

Base: respondents who bought something online – code 1 in S2

P1 [S in each row] **How often do you buy the products/ services of different categories in the following localisations?**
INT.: SHOW THE SCREEN
SCRIPTER: SHOW ONLY PRODUCTS/ SERVICES MENTIONED IN Q03
In row:
1. Groceries
2. Cosmetics
3. Clothing
4. Footwear
5. Leather accessories
6. Small household appliances (vacuum cleaners, dryers, irons, food processors etc.)
7. Big household appliances (washing machines, dishwashers, ovens etc.)
8. TV sets
9. Computers
10. Furniture
11. Interior design articles
12. Garden articles
13. Sport articles
14. Books
15. Music (e.g. Spotify, Tidal)
16. Payable TV (Netflix, VOD, Showmax etc.)

17. Games (for PCs, consoles)
18. Articles for children
19. Toys
20. Dishes takeaway
21. Tickets (travels)
22. Entertainment tickets (concerts, cinema)
23. Accommodation
24. Services, e.g. pre-paid cards, telecommunication services

In columns:
1. Specialised online stores offering only products of one brand
2. Online stores offering products of many brands
3. Allegro
4. OLX
5. Ali Express
6. Stores in shopping centres
7. Stores out of shopping centres
8. Marketplace, street market, door-to-door retail

1. Once a week or more often
2. 2-3 times a month
3. Once a month
4. A few times a year
5. 1-2 times a year
6. More seldom
7. Never

Base: respondents who bought something online – code 1 in S2
P19 [S]
Please compare your average expenditures on online shopping and on shopping in stationary stores. Which of the following statements describes your situation the best?
1. I spend decidedly more on online shopping than on shopping in stationary stores
2. I spend a little bit more on online shopping than on shopping in stationary retail
3. I spend about the same on online shopping and on shopping in stationary retail
4. I spend a little bit more on shopping in stationary retail than on online shopping
5. I spend decidedly more on shopping in stationary retail than on online shopping

Base: all respondents
P20 [S in each row]
To what extent do you agree or do not agree with the following statements considering shopping?
INK.: SHOW THE SCREEN
In rows:
1. I do the shopping online to make a good impression on friends thanks to a cool purchase
2. Shopping online is very fashionable now
3. Shopping online is for me the most convenient way to buy different services and products
4. The Internet serves me as a source of information first of all and I do the shopping in stationary stores in most cases
5. I watch products firstly in a stationary store very often and then I buy them online
6. I am not an enthusiast of online shopping, I prefer traditional shopping
7. I like doing the shopping neither online nor in a traditional way
8. I prefer shopping in stationary stores – you can go outside of home at least and take some exercise

In columns:
1. I do not agree at all
2. Rather I do not agree
3. Neither I agree, nor I do not agree
4. Rather I agree
5. I agree completely

Base: all respondents

P22 [S in each row]

Now, I am reading out some statements describing buying behaviours of different people. After each of statements, please tell me, to what extent do you agree or do not agree with each statement.

INT: SHOW THE SCREEN AND READ OUT

SCRIPTER ROTATION

In rows:

1. I often have an unexplainable urge, a sudden and spontaneous desire, to go and buy sometimes in a store
2. At times, I have felt somewhat guilty after buying a product
3. There are times when I have a strong urge to buy
4. I sometimes feel that something inside of me pushed me to go shopping
5. Often I buy something just because it is cheap
6. Often I buy something because simply I feel like buying
7. Often I have a feeling that I absolutely must have an item
8. There are some things I buy that I do not show to anybody for fear of being perceived as irrational in my buying behaviour
9. Often I ask myself after a purchase of an item if the purchase was really so important
10. As soon as I walk down streets or I enter a shopping centre, I have an irresistible urge to go into a shop to buy something
11. When I have money, I cannot help but spend part of the whole of it
12. I am rather free-spending
13. I am one of those people who often responds to direct mail offers/ websites of online stores
14. For me, shopping is a way of facing the stress of my daily life and of relaxing
15. I have often bought something which I do not use at all
16. I have often bought a product that I did not need, while knowing I had very little money left

In columns:

1. I do not agree
2. Rather I do not agree
3. Rather I agree
4. I totally agree
