# Supplementary material for: Compulsive and compensative buying among online shoppers: An empirical study
Source: PLoS One. 2021 Jun 3;16(6):e0252563. doi: 10.1371/journal.pone.0252563 (PMC8174690; doi:10.1371/journal.pone.0252563)
Supplement: Attachment — Submitted filename: Response_to_Reviewer_2.pdf [file pone.0252563.s003.pdf]

No.	Comments of Reviewer 2	Answer of the author
1		Thank you very much for the inspiring review, your comments and suggestions of improvements
2	Although the authors refer to the prevalence of online shopping in studies carried out in different countries, the importance of this phenomenon at a global level requires the inclusion of some other recent bibliographic references from studies carried out in Asian countries (where its high prevalence is reported). Specifically, some bibliographic reference could be added in lines 72-74, where it is said that: "shopping online is an important universal factor of compulsive buying independent of cultural conditions".	We completed the paper with the results coming from studies conducted in the Asian countries. We introduced the information in the section "Prevalence" + biographic information in the Introduction at the end of lines 72-74.
3	Specify to which scale the authors refer in line 132	Corrected; It should be written in capital letters (line 133)
4	Justify the extensive exposition in the introduction of the variables self-esteem and materialism as factors "strengthening susceptibility to compulsive buying", when the authors do not include them as variables to be analyzed in this research (lines 135-167)	Indeed, the section is too extensive. We limited the section to a few sentences (lines 136-156).
5	It would be convenient to detail more the characteristic of the sample (sex, age, …). It is only indicated that "the study was carried out on the statistically representative sample of 1,000 Poles aged 15 years old and over" (lines 296-297)	We attached the section including the detailed structure of the sample (line 3090
6	It is necessary to refer to the differences between different measures of compulsive buying in the paragraph that presents the self-report used for the assessment of compulsive buying (see lines 303-309)	ADDED (lines 311-327)
7	It would be advisable to indicate which statistical analyses are going to be applied before presenting the results	ADDED in the end of the section (lines 366-373)
8	Include the reference to the various tables of results (tables 1,2 and 3 – lines 391, 442, 513)	DONE, ADDED INFORMATION ABOUT THE SOURCE
9	We suggest to include the description of the statements that evaluate the attitudes towards shopping online in the material section, not in the results section (line 444), as it has been done with the description of the German Compulsive Buying Indicator (line 303)	DONE, THE DESCRIPTION IS MOVED TO THE SECTION OF MATERIALS (lines 337-365)
10	It would be interesting if the authors, based on the findings, could suggest some special guidelines for action and/ or what implications may have for the design of future prevention/ intervention programs for compulsive online shopping	ADDED AT THE END OF THE PAPER (lines 745-762)
11	We recommend to review the inclusion of references in the text, since on many occasions the bibliographic references are duplicated	IMPROVED
